# Crystal structure of 2-(3-fluoro­phen­yl)-5-iodo-3-methyl­sulfinyl-1-benzo­furan

**DOI:** 10.1107/S1600536814022569

**Published:** 2014-10-18

**Authors:** Hong Dae Choi, Uk Lee

**Affiliations:** a Department of Chemistry, Dongeui University, San 24 Kaya-dong, Busanjin-gu, Busan 614-714, Republic of Korea; b Department of Chemistry, Pukyong National University, 599-1 Daeyeon 3-dong, Nam-gu, Busan 608-737, Republic of Korea

**Keywords:** crystal structure, benzo­furan, 3-fluoro­phen­yl, C—H⋯O hydrogen bonds, I⋯O contacts

## Abstract

In the title compound, C_15_H_10_FIO_2_S, the dihedral angle between the planes of the benzo­furan ring system [r.m.s. deviation = 0.015 (2) Å] and the 3-fluoro­phenyl ring is 29.63 (7)°. In the crystal, mol­ecules are linked into inversion dimers along the *b*-axis direction by two different pairs of C—H⋯O hydrogen bonds and I⋯O [3.228 (1) Å] contacts.

## Related literature   

For a related structure and background to benzo­furan derivatives, see: Choi & Lee (2014 ▶). For further synthetic details, see: Choi *et al.* (1999 ▶). For a review of halogen bonding, see: Politzer *et al.* (2007 ▶).
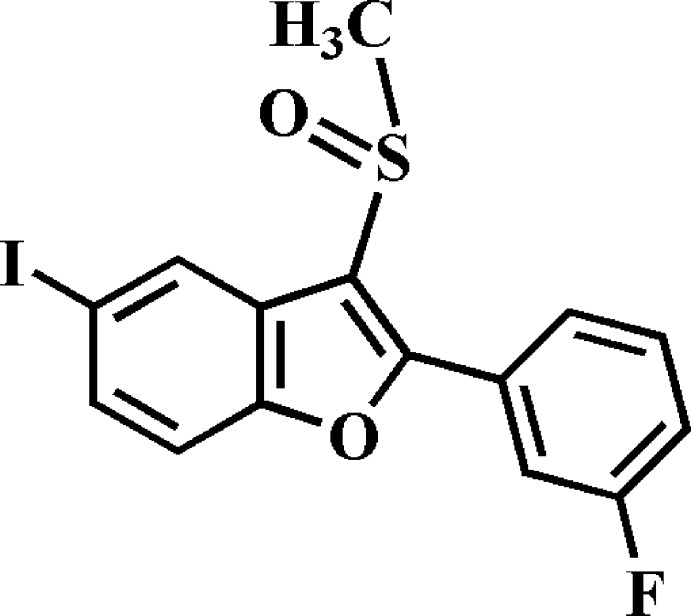


## Experimental   

### Crystal data  

C_15_H_10_FIO_2_S
*M* *_r_* = 400.19
Triclinic, 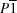

*a* = 8.1348 (3) Å
*b* = 8.6378 (3) Å
*c* = 10.8350 (4) Å
α = 86.063 (1)°
β = 82.088 (1)°
γ = 66.408 (1)°
*V* = 690.99 (4) Å^3^
*Z* = 2
Mo *K*α radiation
μ = 2.48 mm^−1^
*T* = 173 K
0.45 × 0.28 × 0.11 mm

### Data collection  

Bruker SMART APEXII CCD diffractometer
Absorption correction: multi-scan (*SADABS*; Bruker, 2009 ▶) *T*_min_ = 0.496, *T*_max_ = 0.746
12585 measured reflections
3450 independent reflections
3226 reflections with *I* > 2σ(*I*)
*R*_int_ = 0.028

### Refinement  

*R*[*F* ^2^ > 2σ(*F* ^2^)] = 0.021
*wR*(*F* ^2^) = 0.054
*S* = 1.05
3450 reflections
182 parameters
H-atom parameters constrained
Δρ_max_ = 0.71 e Å^−3^
Δρ_min_ = −0.59 e Å^−3^

### 

Data collection: *APEX2* (Bruker, 2009 ▶); cell refinement: *SAINT* (Bruker, 2009 ▶); data reduction: *SAINT*; program(s) used to solve structure: *SHELXS97* (Sheldrick, 2008 ▶); program(s) used to refine structure: *SHELXL97* (Sheldrick, 2008 ▶); molecular graphics: *ORTEP-3 for Windows* (Farrugia, 2012 ▶) and *DIAMOND* (Brandenburg, 1998 ▶); software used to prepare material for publication: *SHELXL97*.

## Supplementary Material

Crystal structure: contains datablock(s) I. DOI: 10.1107/S1600536814022569/qm2109sup1.cif

Structure factors: contains datablock(s) I. DOI: 10.1107/S1600536814022569/qm2109Isup2.hkl

Supporting information file. DOI: 10.1107/S1600536814022569/qm2109Isup3.cml

**Fig. 1.**
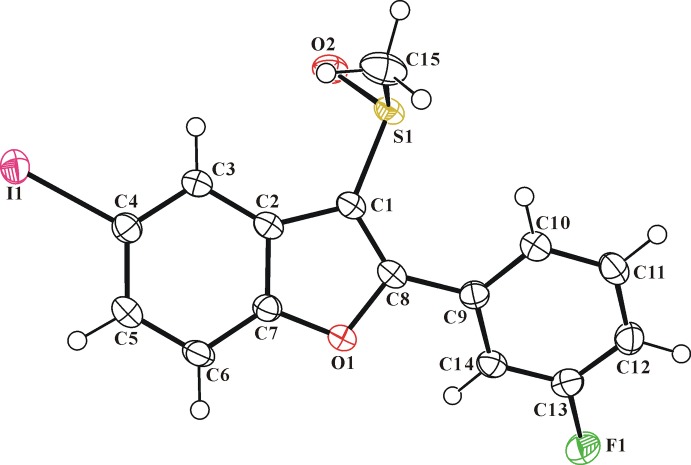
The molecular structure of the title compound with the atom-numbering scheme. Displacement ellipsoids are drawn at the 50% probability level. H atoms are presented as small spheres of arbitrary radius.

**Fig. 2.**
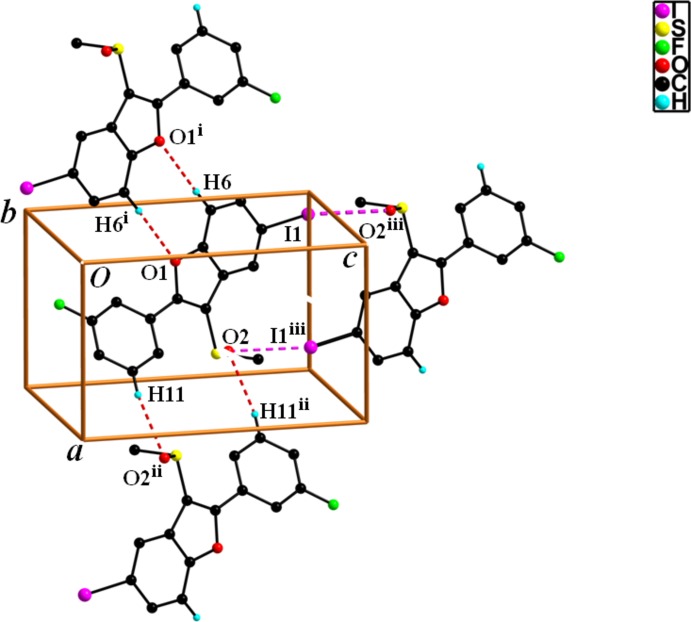
A view of the C—H···O and I···O interactions (dotted lines) in the crystal structure of the title compound. H atoms non-participating in hydrogen-bonding were omitted for clarity. [Symmetry codes: (i) -x, -y + 2, -z + 1; (ii) -x + 2, -y + 1, -z; (iii) -x + 1, -y + 2, -z + 2.]

. DOI: 10.1107/S1600536814022569/qm2109fig1.tif
The mol­ecular structure of the title compound with the atom-numbering scheme. Displacement ellipsoids are drawn at the 50% probability level. H atoms are presented as small spheres of arbitrary radius.

x y z x y z x y z . DOI: 10.1107/S1600536814022569/qm2109fig2.tif
A view of the C—H⋯O and I⋯O inter­actions (dotted lines) in the crystal structure of the title compound. H atoms non-participating in hydrogen-bonding were omitted for clarity. [Symmetry codes: (i) −*x*, −*y* + 2, −*z* + 1; (ii) −*x* + 2, −*y* + 1, −*z*; (iii) −*x* + 1, −*y* + 2, −*z* + 2.]

CCDC reference: 1029138

Additional supporting information:  crystallographic information; 3D view; checkCIF report

## Figures and Tables

**Table 1:** Hydrogen-bond geometry (, )

*D*H*A*	*D*H	H*A*	*D* *A*	*D*H*A*
C6H6O1^i^	0.95	2.57	3.520(2)	177
C11H11O2^ii^	0.95	2.55	3.372(2)	145

Symmetry codes: (i) 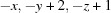; (ii) 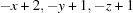.

## supplementary crystallographic information

### S1. Comment

As part of our continuing program for benzofuran derivatives (Choi & Lee, 2014), we report herein on the crystal structure of the title compound.

In the title molecule (Fig. 1), the benzofuran unit is essentially planar, with a mean deviation of 0.015 (2) Å from the least-squares plane defined by the nine constituent atoms. The 3-fluorophenyl ring is essentially planar, with a mean deviation of 0.002 (1) Å from the least-squares plane defined by the six constituent atoms. The dihedral angle formed by the benzofuran ring and the 3-fluorophenyl ring is 29.63 (7)°. In the crystal structure (Fig. 2), molecules are linked into inversion-related dimers along *b*-axis direction by two different pairs of C—H···O hydrogen bonds (Table 1) and I···O halogen-bondings (Politzer *et al.*, 2007) between the iodine and the O atom of the sulfinyl group [I1···O2^iii^ = 3.228 (2) Å, C4—I1···O2^iii^ = 162.99 (6)°, symmetry code: (iii) - *x* + 1, - *y* + 2, - *z* + 2],

### S2. Experimental

The starting material 2-(3-fluorophenyl)-5-iodo-3-methylsulfanyl-1-benzofuran was prepared by literature method (Choi *et al.*, 1999). 3-Chloroperoxybenzoic acid (77%, 224 mg, 1.0 mmol) was added in small portions to a stirred solution of 2-(3-fluorophenyl)-5-iodo-3-methylsulfanyl-1-benzofuran (346 mg, 0.9 mmol) in dichloromethane (30 ml) at 273 K. After being stirred at room temperature for 8h, the mixture was washed with saturated sodium bicarbonate solution (2 X 10 ml) and the organic layer was separated, dried over magnesium sulfate, filtered and concentrated at reduced pressure. The residue was purified by column chromatography (hexane–ethyl acetate, 1:2 *v*/*v*) to afford the title compound as a colorless solid [yield 73% (263 mg); m.p. 453–454 K; *R*_f_ = 0.55 (hexane–ethyl acetate, 1:2 *v*/*v*)]. Single crystals suitable for X-ray diffraction were prepared by slow evaporation of a solution of the title compound (26 mg) in ethyl acetate (25 ml) at room temperature.

### S3. Refinement

All H atoms were positioned geometrically and refined using a riding model, with C—H = 0.95 Å for aryl, and 0.98 Å for methyl H atoms, respectively. *U*_iso_ (H) = 1.2*U*_eq_ (C) for aryl and 1.5*U*_eq_ (C)) for methyl H atoms. The positions of methyl hydrogens were optimized using the SHELXL-97's command AFIX 137 (Sheldrick, 2008).

### Crystal data

**Table:**

C_15_H_10_FIO_2_S	*Z* = 2
*M**_r_* = 400.19	*F*(000) = 388
Triclinic, *P*1	*D*_x_ = 1.923 Mg m^−^^3^
Hall symbol: -P 1	Melting point = 454–453 K
*a* = 8.1348 (3) Å	Mo *K*α radiation, λ = 0.71073 Å
*b* = 8.6378 (3) Å	Cell parameters from 8421 reflections
*c* = 10.8350 (4) Å	θ = 2.6–28.4°
α = 86.063 (1)°	µ = 2.48 mm^−^^1^
β = 82.088 (1)°	*T* = 173 K
γ = 66.408 (1)°	Block, colourless
*V* = 690.99 (4) Å^3^	0.45 × 0.28 × 0.11 mm

### Data collection

**Table:**

Bruker SMART APEXII CCD diffractometer	3450 independent reflections
Radiation source: rotating anode	3226 reflections with *I* > 2σ(*I*)
Graphite multilayer monochromator	*R*_int_ = 0.028
Detector resolution: 10.0 pixels mm^-1^	θ_max_ = 28.4°, θ_min_ = 1.9°
φ and ω scans	*h* = −10→10
Absorption correction: multi-scan (*SADABS*; Bruker, 2009)	*k* = −11→11
*T*_min_ = 0.496, *T*_max_ = 0.746	*l* = −14→14
12585 measured reflections	

### Refinement

**Table:**

Refinement on *F*^2^	Primary atom site location: structure-invariant direct methods
Least-squares matrix: full	Secondary atom site location: difference Fourier map
*R*[*F*^2^ > 2σ(*F*^2^)] = 0.021	Hydrogen site location: difference Fourier map
*wR*(*F*^2^) = 0.054	H-atom parameters constrained
*S* = 1.05	*w* = 1/[σ^2^(*F*_o_^2^) + (0.0277*P*)^2^ + 0.2506*P*] where *P* = (*F*_o_^2^ + 2*F*_c_^2^)/3
3450 reflections	(Δ/σ)_max_ = 0.002
182 parameters	Δρ_max_ = 0.71 e Å^−^^3^
0 restraints	Δρ_min_ = −0.59 e Å^−^^3^

### Special details

**Table:**

Experimental. ^1^H NMR (δ p.p.m., CDCl_3_, 400 Hz): 8.59 (s, 1H), 7.70 (dd, J =8.56 and 1.72 Hz, 1H), 7.46-7.62 (m, 3H), 7.36 (d, J =8.88 Hz, 1H), 6.16-7.22 (m, 1H), 3.11 (s, 3H).
Geometry. All esds (except the esd in the dihedral angle between two l.s. planes) are estimated using the full covariance matrix. The cell esds are taken into account individually in the estimation of esds in distances, angles and torsion angles; correlations between esds in cell parameters are only used when they are defined by crystal symmetry. An approximate (isotropic) treatment of cell esds is used for estimating esds involving l.s. planes.
Refinement. Refinement of F^2^ against ALL reflections. The weighted R-factor wR and goodness of fit S are based on F^2^, conventional R-factors R are based on F, with F set to zero for negative F^2^. The threshold expression of F^2^ > 2sigma(F^2^) is used only for calculating R-factors(gt) etc. and is not relevant to the choice of reflections for refinement. R-factors based on F^2^ are statistically about twice as large as those based on F, and R- factors based on ALL data will be even larger.

### Fractional atomic coordinates and isotropic or equivalent isotropic displacement parameters (Å^2^)

**Table:**

	*x*	*y*	*z*	*U*_iso_*/*U*_eq_	
I1	0.171392 (16)	1.149394 (16)	1.025925 (12)	0.03179 (6)	
S1	0.78329 (6)	0.70139 (6)	0.61226 (5)	0.02565 (10)	
F1	0.44813 (19)	0.69674 (18)	0.05731 (12)	0.0428 (3)	
O1	0.29753 (17)	0.86217 (16)	0.49979 (13)	0.0257 (3)	
O2	0.81435 (19)	0.83024 (18)	0.67958 (15)	0.0322 (3)	
C1	0.5521 (2)	0.7849 (2)	0.59123 (18)	0.0236 (4)	
C2	0.4042 (2)	0.8963 (2)	0.67518 (18)	0.0229 (3)	
C3	0.3844 (2)	0.9583 (2)	0.79446 (18)	0.0245 (4)	
H3	0.4851	0.9312	0.8393	0.029*	
C4	0.2108 (2)	1.0612 (2)	0.84450 (18)	0.0252 (4)	
C5	0.0602 (3)	1.1041 (2)	0.77957 (19)	0.0279 (4)	
H5	−0.0560	1.1753	0.8173	0.033*	
C6	0.0793 (3)	1.0437 (2)	0.6614 (2)	0.0280 (4)	
H6	−0.0209	1.0718	0.6158	0.034*	
C7	0.2528 (2)	0.9398 (2)	0.61297 (18)	0.0240 (4)	
C8	0.4815 (2)	0.7686 (2)	0.48808 (18)	0.0239 (4)	
C9	0.5539 (2)	0.6727 (2)	0.37323 (18)	0.0246 (4)	
C10	0.7027 (3)	0.5184 (2)	0.36961 (19)	0.0284 (4)	
H10	0.7611	0.4757	0.4422	0.034*	
C11	0.7657 (3)	0.4272 (3)	0.2604 (2)	0.0298 (4)	
H11	0.8680	0.3228	0.2583	0.036*	
C12	0.6806 (3)	0.4872 (3)	0.1544 (2)	0.0310 (4)	
H12	0.7223	0.4250	0.0794	0.037*	
C14	0.4675 (3)	0.7348 (2)	0.26611 (18)	0.0259 (4)	
H14	0.3661	0.8396	0.2666	0.031*	
C13	0.5339 (3)	0.6395 (3)	0.16068 (19)	0.0296 (4)	
C15	0.7795 (3)	0.5423 (3)	0.7268 (2)	0.0399 (5)	
H15A	0.8990	0.4863	0.7554	0.060*	
H15B	0.7483	0.4587	0.6898	0.060*	
H15C	0.6893	0.5949	0.7976	0.060*	

### Atomic displacement parameters (Å^2^)

**Table:**

	*U*^11^	*U*^22^	*U*^33^	*U*^12^	*U*^13^	*U*^23^
I1	0.02741 (8)	0.03722 (9)	0.02882 (8)	−0.01142 (6)	0.00213 (5)	−0.00751 (6)
S1	0.01711 (19)	0.0255 (2)	0.0331 (2)	−0.00688 (17)	−0.00367 (17)	−0.00041 (18)
F1	0.0462 (8)	0.0463 (8)	0.0316 (7)	−0.0101 (6)	−0.0147 (6)	−0.0038 (6)
O1	0.0192 (6)	0.0277 (7)	0.0269 (7)	−0.0050 (5)	−0.0048 (5)	−0.0025 (5)
O2	0.0279 (7)	0.0310 (7)	0.0421 (8)	−0.0142 (6)	−0.0108 (6)	0.0011 (6)
C1	0.0189 (8)	0.0222 (8)	0.0287 (9)	−0.0072 (7)	−0.0034 (7)	0.0005 (7)
C2	0.0187 (8)	0.0212 (8)	0.0283 (9)	−0.0078 (7)	−0.0032 (7)	0.0020 (7)
C3	0.0205 (8)	0.0259 (9)	0.0272 (9)	−0.0089 (7)	−0.0043 (7)	0.0010 (7)
C4	0.0232 (8)	0.0245 (9)	0.0279 (9)	−0.0100 (7)	−0.0014 (7)	−0.0001 (7)
C5	0.0204 (8)	0.0254 (9)	0.0341 (10)	−0.0059 (7)	−0.0007 (7)	−0.0007 (8)
C6	0.0184 (8)	0.0292 (9)	0.0340 (10)	−0.0059 (7)	−0.0063 (7)	0.0000 (8)
C7	0.0218 (8)	0.0235 (8)	0.0254 (9)	−0.0074 (7)	−0.0045 (7)	0.0004 (7)
C8	0.0181 (8)	0.0215 (8)	0.0295 (9)	−0.0060 (7)	−0.0019 (7)	0.0019 (7)
C9	0.0228 (8)	0.0258 (9)	0.0280 (9)	−0.0127 (7)	−0.0025 (7)	−0.0013 (7)
C10	0.0248 (9)	0.0283 (9)	0.0305 (10)	−0.0085 (7)	−0.0042 (7)	−0.0010 (8)
C11	0.0225 (9)	0.0270 (9)	0.0363 (11)	−0.0066 (7)	−0.0002 (8)	−0.0030 (8)
C12	0.0284 (9)	0.0331 (10)	0.0318 (10)	−0.0128 (8)	0.0008 (8)	−0.0076 (8)
C14	0.0224 (8)	0.0254 (9)	0.0307 (9)	−0.0101 (7)	−0.0043 (7)	0.0009 (7)
C13	0.0291 (9)	0.0342 (10)	0.0283 (10)	−0.0148 (8)	−0.0061 (8)	0.0000 (8)
C15	0.0357 (11)	0.0310 (11)	0.0560 (14)	−0.0147 (9)	−0.0186 (10)	0.0156 (10)

### Geometric parameters (Å, º)

**Table:**

I1—C4	2.0937 (19)	C6—C7	1.385 (3)
I1—O2^i^	3.2282 (16)	C6—H6	0.9500
S1—O2	1.4906 (15)	C8—C9	1.460 (3)
S1—C1	1.7660 (18)	C9—C10	1.396 (3)
S1—C15	1.794 (2)	C9—C14	1.402 (3)
F1—C13	1.360 (2)	C10—C11	1.386 (3)
O1—C7	1.371 (2)	C10—H10	0.9500
O1—C8	1.380 (2)	C11—C12	1.385 (3)
C1—C8	1.364 (3)	C11—H11	0.9500
C1—C2	1.447 (2)	C12—C13	1.376 (3)
C2—C7	1.392 (3)	C12—H12	0.9500
C2—C3	1.397 (3)	C14—C13	1.370 (3)
C3—C4	1.389 (2)	C14—H14	0.9500
C3—H3	0.9500	C15—H15A	0.9800
C4—C5	1.404 (3)	C15—H15B	0.9800
C5—C6	1.382 (3)	C15—H15C	0.9800
C5—H5	0.9500		
			
C4—I1—O2^i^	162.99 (6)	C1—C8—C9	135.08 (17)
O2—S1—C1	107.27 (8)	O1—C8—C9	114.36 (16)
O2—S1—C15	105.59 (11)	C10—C9—C14	119.72 (18)
C1—S1—C15	97.77 (10)	C10—C9—C8	121.22 (18)
C7—O1—C8	106.51 (14)	C14—C9—C8	119.02 (17)
C8—C1—C2	107.22 (16)	C11—C10—C9	120.19 (19)
C8—C1—S1	126.32 (14)	C11—C10—H10	119.9
C2—C1—S1	126.23 (14)	C9—C10—H10	119.9
C7—C2—C3	119.44 (16)	C12—C11—C10	120.41 (19)
C7—C2—C1	104.72 (16)	C12—C11—H11	119.8
C3—C2—C1	135.82 (17)	C10—C11—H11	119.8
C4—C3—C2	116.96 (17)	C13—C12—C11	118.20 (18)
C4—C3—H3	121.5	C13—C12—H12	120.9
C2—C3—H3	121.5	C11—C12—H12	120.9
C3—C4—C5	122.53 (18)	C13—C14—C9	117.96 (18)
C3—C4—I1	118.63 (14)	C13—C14—H14	121.0
C5—C4—I1	118.81 (13)	C9—C14—H14	121.0
C6—C5—C4	120.71 (17)	F1—C13—C14	118.31 (18)
C6—C5—H5	119.6	F1—C13—C12	118.15 (18)
C4—C5—H5	119.6	C14—C13—C12	123.5 (2)
C5—C6—C7	116.21 (18)	S1—C15—H15A	109.5
C5—C6—H6	121.9	S1—C15—H15B	109.5
C7—C6—H6	121.9	H15A—C15—H15B	109.5
O1—C7—C6	124.79 (17)	S1—C15—H15C	109.5
O1—C7—C2	111.03 (15)	H15A—C15—H15C	109.5
C6—C7—C2	124.15 (18)	H15B—C15—H15C	109.5
C1—C8—O1	110.51 (16)		
			
O2—S1—C1—C8	141.12 (17)	C3—C2—C7—C6	0.5 (3)
C15—S1—C1—C8	−109.83 (19)	C1—C2—C7—C6	178.84 (18)
O2—S1—C1—C2	−32.64 (19)	C2—C1—C8—O1	0.0 (2)
C15—S1—C1—C2	76.41 (18)	S1—C1—C8—O1	−174.78 (13)
C8—C1—C2—C7	−0.4 (2)	C2—C1—C8—C9	−177.5 (2)
S1—C1—C2—C7	174.33 (15)	S1—C1—C8—C9	7.7 (3)
C8—C1—C2—C3	177.6 (2)	C7—O1—C8—C1	0.5 (2)
S1—C1—C2—C3	−7.7 (3)	C7—O1—C8—C9	178.56 (16)
C7—C2—C3—C4	0.2 (3)	C1—C8—C9—C10	29.0 (3)
C1—C2—C3—C4	−177.6 (2)	O1—C8—C9—C10	−148.40 (17)
C2—C3—C4—C5	−0.6 (3)	C1—C8—C9—C14	−153.2 (2)
C2—C3—C4—I1	177.44 (13)	O1—C8—C9—C14	29.3 (2)
O2^i^—I1—C4—C3	−46.0 (3)	C14—C9—C10—C11	0.4 (3)
O2^i^—I1—C4—C5	132.10 (18)	C8—C9—C10—C11	178.10 (18)
C3—C4—C5—C6	0.3 (3)	C9—C10—C11—C12	−0.7 (3)
I1—C4—C5—C6	−177.70 (15)	C10—C11—C12—C13	0.5 (3)
C4—C5—C6—C7	0.3 (3)	C10—C9—C14—C13	0.1 (3)
C8—O1—C7—C6	−178.87 (18)	C8—C9—C14—C13	−177.66 (17)
C8—O1—C7—C2	−0.8 (2)	C9—C14—C13—F1	178.38 (17)
C5—C6—C7—O1	177.14 (18)	C9—C14—C13—C12	−0.3 (3)
C5—C6—C7—C2	−0.7 (3)	C11—C12—C13—F1	−178.69 (18)
C3—C2—C7—O1	−177.65 (16)	C11—C12—C13—C14	0.0 (3)
C1—C2—C7—O1	0.7 (2)		

Symmetry code: (i) −*x*+1, −*y*+2, −*z*+2.

### Hydrogen-bond geometry (Å, º)

**Table:**

*D*—H···*A*	*D*—H	H···*A*	*D*···*A*	*D*—H···*A*
C6—H6···O1^ii^	0.95	2.57	3.520 (2)	177
C11—H11···O2^iii^	0.95	2.55	3.372 (2)	145

Symmetry codes: (ii) −*x*, −*y*+2, −*z*+1; (iii) −*x*+2, −*y*+1, −*z*+1.
