# Supplementary figures and images for: Crystal structure of 2-(3-fluoro­phen­yl)-5-iodo-3-methyl­sulfinyl-1-benzo­furan
Source: Acta Crystallogr Sect E Struct Rep Online. 2014 Oct 18;70(Pt 11):o1169. doi: 10.1107/S1600536814022569 (PMC4257291; doi:10.1107/S1600536814022569)

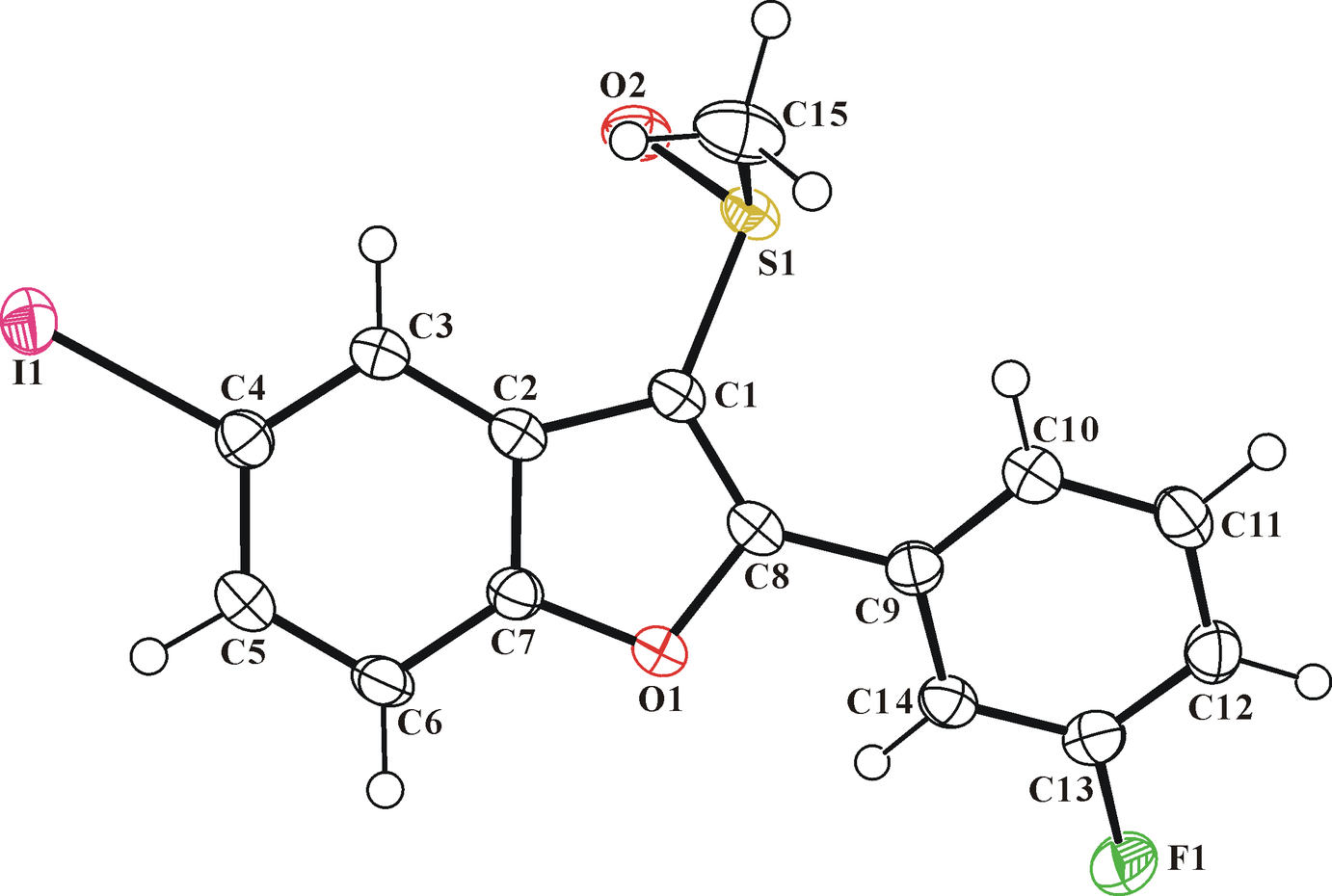

Supplement: Supplementary file 4 [file e-70-o1169-fig1.tif]

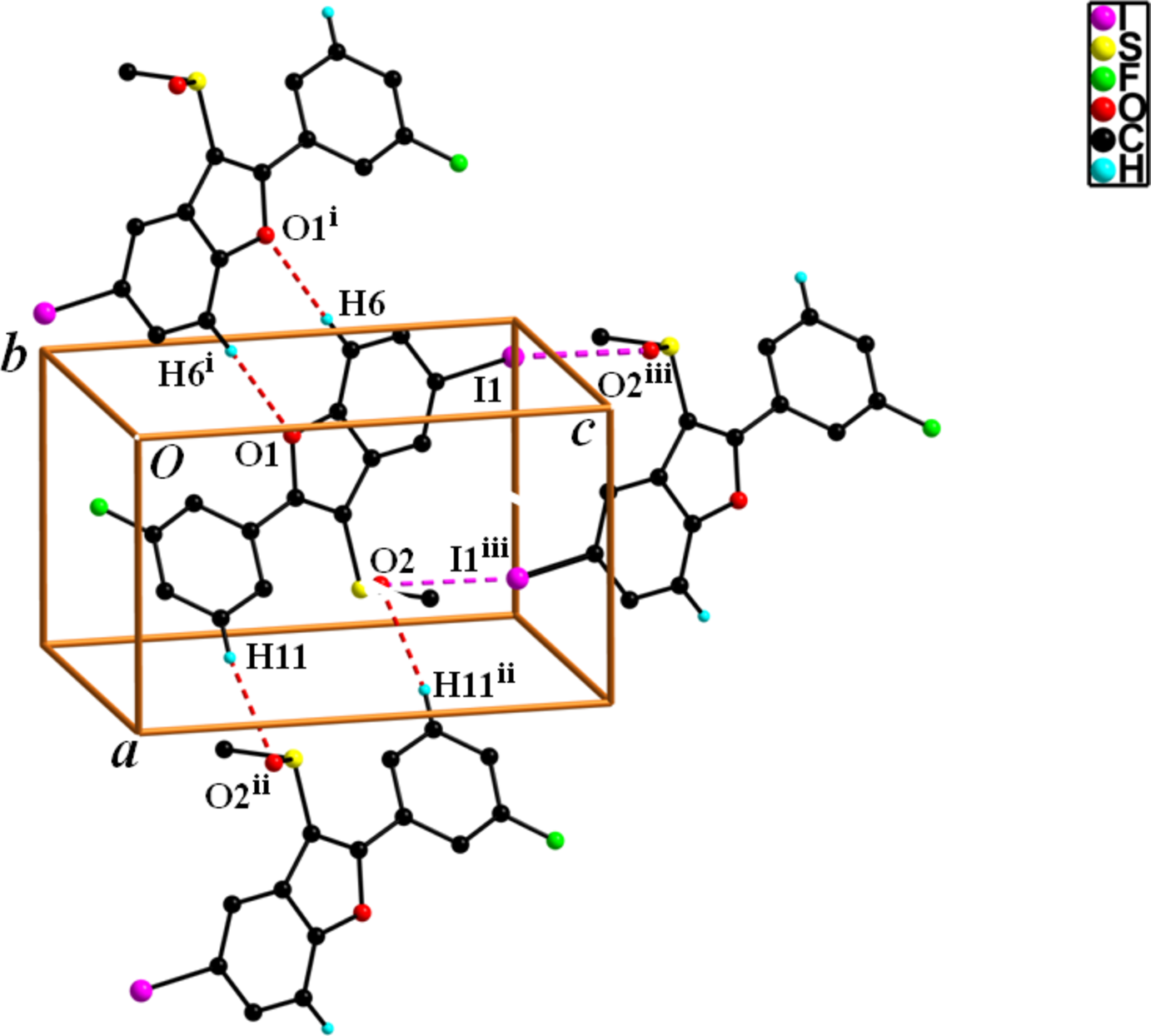

Supplement: Supplementary file 5 [file e-70-o1169-fig2.tif]
